# Role of Viral and Host microRNAs in Immune Regulation of Epstein-Barr Virus-Associated Diseases

**DOI:** 10.3389/fimmu.2020.00367

**Published:** 2020-03-03

**Authors:** Hisashi Iizasa, Hyoji Kim, Andy Visi Kartika, Yuichi Kanehiro, Hironori Yoshiyama

**Affiliations:** Department of Microbiology, Faculty of Medicine, Shimane University, Shimane, Japan

**Keywords:** microRNAs, herpes virus, immune evasion, BART miRNA, BHRF miRNA

## Abstract

Epstein-Barr virus (EBV) is an oncogenic human herpes virus that was discovered in 1964. Viral non-coding RNAs, such as *Bam*HI-A rightward fragment-derived microRNAs (BART miRNAs) or *Bam*HI-H rightward fragment 1-derived miRNAs (BHRF1 miRNA) in EBV-infected cells have been recently reported. Host miRNAs are also upregulated upon EBV infection. Viral and host miRNAs are important in maintaining viral infection and evasion of host immunity. Although miRNAs in EBV-infected cells often promote cell proliferation by targeting apoptosis or cell cycle, this review focuses on the regulation of the recognition of the host immune system. This review firstly describes the location and organization of two clusters of viral miRNAs, then describes evasion from host immune surveillance systems by modulating viral gene expression or inhibiting innate and acquired immunity by viral miRNAs as well as host miRNAs. Another topic is the enigmatic depletion of viral miRNAs in several types of EBV-infected tumor cells. Finally, this review introduces the strong correlation of nasopharyngeal cancer cases with a newly identified single nucleotide polymorphism that enhances BART miRNA promoter activity.

## Introduction

Epstein-Barr virus (EBV) is a double-stranded DNA virus that belongs to the Gammaherpesvirus subfamily and was discovered in a Burkitt's lymphoma (BL) cell (1). EBV primarily infects B cells via the high-affinity receptor CD21; it also infects CD21-negative T cells, natural killer (NK) cells, and epithelial cells using low-affinity receptors (2). EBV causes the primary acute disease “infectious mononucleosis” in adolescents (3). Following a primary infection in B lymphocytes or epithelial cells, EBV establishes a chronic infection known as latent infection.

The two infection cycles that enable successful propagation of the EBV progeny viruses are lytic and latent infection. During lytic infection, all the viral genes are expressed and the viral genome is rapidly replicated. In contrast, latent infection involves the restricted expression of a number of viral genes. Here, EBV evades host immune surveillance and the copy number of DNA in the viral daughter cells are maintained by synchronous duplication of viral and host genomes. A small subset of viral genes and microRNAs (miRNAs) expressed during the latent infection maintain viral episomes and stimulate host cell proliferation. EBV propagates viral genomes together with host cells during latent infection.

Host cell proliferation associated with latent EBV infection induces malignancies, such as BL, Hodgkin's lymphoma (HL), EBV-positive diffuse large B-cell lymphoma (DLBCL), extranodal NK/T-cell lymphoma-nasal type (ENKL), nasopharyngeal carcinoma (NPC), and EBV-associated gastric carcinoma. EBV also causes the severe infectious disease called chronic active EBV infection (3–6).

A miRNA is a non-coding single-stranded RNA comprising 20–22 bases that regulates post-transcriptional gene expression. More than 60% of protein-coding genes are regulated by miRNAs in mammals (7). miRNAs are present in both eukaryotic and viral genomes, such as the EBV genome (8). Viral miRNAs are incorporated into the RNA-induced silencing complex and this miRNA complex interacts with the 3′ untranslated region of host and viral mRNAs. This suppresses the expression of target gene(s) via translational repression or mRNA degradation (9). Viral miRNAs suppress target genes in the EBV and host genomes to maintain latent EBV infection, evade the host immune surveillance system, and promote tumorigenic growth of infected cells among other functions (10).

Here we discuss the role of EBV-encoded miRNAs in maintaining latent and lytic infection along with the function of host and viral miRNAs in regulating immune responses in EBV-associated diseases.

## EBV-Encoded miRNAs (EBV miRNAs)

EBV-encoded *Bam*H I-A rightward transcripts (BARTs) are alternatively spliced non-coding RNAs abundantly expressed during latent infection (11). B95-8 is a representative EBV strain with a deletion in a major portion of BART. This strain can transform B lymphocytes and produce progeny viral particles in abundance (12). Because previous EBV studies have mostly based on the *in vitro* immortalizing assay of primary B lymphocytes, the role of BART in the viral life cycle could only be studied after the discovery of BART miRNAs.

**Figure 1:**
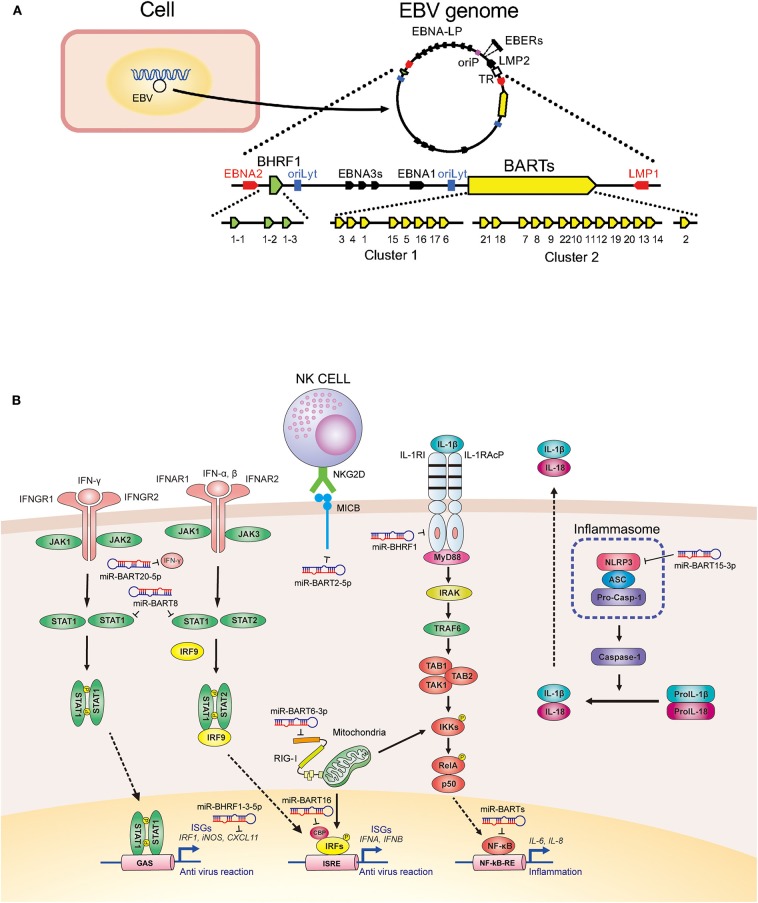
Expression and function of Epstein-Barr virus-derived microRNAs (EBV-derived miRNAs). **(A)** Genomic organization of EBV miRNAs. **(B)** Signaling cascades involved in EBV miRNA-mediated repression of host innate immunity.

Wild-type EBV contains 44 BART miRNAs that are separated by an intron resulting in BART miRNA clusters 1 and 2 (13). Double-stranded RNAs transcribed from the EBV genome are processed by the host miRNA machinery to produce viral miRNAs (9). BART miRNA cluster 1 contains primary transcripts for eight miRNA (pri-miRNAs), namely pri-miR-BART1, 3–6, and 15–17. BART miRNA cluster 2 encodes 13 pri-miRNAs, including pri-miR-BART21, 18, 7, 8, 9, 22, 10, 11, 12, 19, 20, 13, and 14. The deletion in B95-8 encompasses pri-miR-BART15 to the 13 pri-miRNAs in cluster 2 (13) (Figure 1A).

The gene for *Bam*H I-H right fragment 1 (BHRF1) encodes for three pri-miRNAs called pri-miR-BHRF1-1, -BHRF1-2, and -BHRF1-3. BHRF1 miRNAs are expressed during lytic infection, inhibit apoptosis, and favor proliferation of infected cells to enable the early phase of viral propagation (14) (Figure 1A).

Since viruses infect eukaryotic organisms to proliferate, viral miRNAs regulate host cell function and viral life cycle, including viral infection and development of viral progeny (9). EBV miRNAs are more strongly expressed in ENKL and NPC/EBV-associated gastric carcinoma as compared to B cell lymphomas (15).

## Modulation of Viral Gene Expression by EBV miRNAs

EBV miRNAs regulate viral antigen expression (10). miR-BART1-5p, miR-BART16-5p, miR-BART17-5p, and miR-BART9-5p suppress the increase in expression of the highly immunogenic viral latent membrane protein 1 (LMP1) (16, 17). miR-BART22 inhibits the expression of the immunogenic latent membrane protein 2A (18). miR-BART20-5p represses the synthesis of viral transcription factors *Bam*H I-Z leftward reading frame 1 (BZLF1) and *Bam*H I-R leftward reading frame 1 that enable switching between latent and lytic EBV infection (19). miR-BART2-5p hinders the production of viral DNA polymerase *Bam*H I-A leftward reading frame 5 during the latent phase to prevent the transition to lytic replication (13, 20). Therefore, viral strains deficient in all BART miRNAs cannot maintain latent infection since they strongly express BZLF1 that allows the switch to lytic replication (21).

## Regulation of Host Immunity by EBV miRNAs

### Suppression of Host Innate Immunity by EBV miRNAs

EBV miRNAs target viral and host genes involved in innate immunity (Figure 1B and Table 1) (10).

**Table 1:** EBV miRNAs targeting host immune related genes.

**miRNA**	**Host targeting genes**	**Target**	**References**	**EBV infected cells**
**BART miRNAs cluster 1**				
miR-BART1-3p	IL12B	Adaptive immunity	(22)	LCL
miR-BART1-5p	LY75	Adaptive immunity	(23)	LCL
miR-BART1-5p, 3p	IFI30	Adaptive immunity	(24)	LCL
miR-BART15-3p	NLRP3	Innate immunity	(25)	BL
miR-BART16-5p	CREBBP	Innate immunity	(26)	BL, EBVaGC
miR-BART17-5p	TAP2	Adaptive immunity	(24)	LCL
miR-BART6-3p	RIG-I	Innate immunity	(27)	NPC
**BART miRNAs cluster 2**				
miR-BART18-5p	MAP3K2	BCR signals, Adaptive Immunity	(28)	BL
miR-BART8-5p, 3p	STAT1	Innate immunity	(29)	NKTL
miR-BART22-3p	IL12B	Adaptive immunity	(22)	LCL
miR-BART10-3p	IL12B	Adaptive immunity	(22)	LCL
miR-BART20-5p	IFNG	Innate immunity	(29)	NKTL
**Others**				
miR BARTs	NF-kB signal	Innate immunity	(30)	NPC
miR-BART2-5p	MICB	Immunoreaction against NK cell	(31)	LCL
miR-BART2-5p	IL12B	Adaptive immunity	(22, 32)	LCL
miR-BART2-5p	CSTB	Adaptive immunity	(24)	LCL
miR-BART2-5p	LGMN	Adaptive immunity	(24)	LCL
miR-BHRF1	IL-1R1	Innate immunity	(33)	LCL
miR-BHRF1-2-5p	MALT1	BCR signals, Adaptive Immunity	(34)	LCL, DLBL
miR-BHRF1-2-5p	GRB2	BCR signals, Adaptive Immunity	(34)	LCL, DLBL
miR-BHRF1-2-5p	PAG1	BCR signals, Adaptive Immunity	(34)	LCL, DLBL
miR-BHRF1-2-3p	IL12B	Adaptive immunity	(22, 32)	LCL
miR-BHRF1-2-3p	CSTB	Adaptive immunity	(24)	LCL
miR-BHRF1-2-3p	TAP2	Adaptive immunity	(24)	LCL
miR-BHRF1-3-5p	CXCL-11	Innate immunity	(35)	BL, DLBCL

*LCL, Lymphoblastoid cell lines; BL, Burkitt lymphoma; NPC, Nasopharengeal carcinoma; EBVaGC, EBV asspciated gastridc carcinoma; NKTL, NK/T lymphoma; DLBCL, Diffuce large B cell lymphoma*.

During lytic infection, miR-BHRF1-2-5p targets the 3′ untranslated region of the interleukin-1 receptor 1 (IL-1R1) and suppresses IL-1 signaling (33). miR-BHRF1-3-5p in EBV-infected B cells downregulates C-X-C motif chemokine 11 (CXCL-11) that is a downstream effector in interferon gamma (IFN-γ) signaling (35).

miR-BART6-3p targets the retinoic acid-inducible gene-I (RIG-I) (an intracellular receptor for double-stranded RNA), thereby suppressing host innate immune responses (27). miR-BART20-5p and miR-BART8 target IFN-γ and the signal transducer and activator of transcription 1 (STAT1), respectively, ultimately suppressing cellular immunity against tumor cells (29). miR-BART16 targets the cAMP response element-binding protein-binding protein (CBP) (a transcriptional coactivator for type I IFN signaling) in EBV-infected B cells and epithelial cells to inhibit IFN signaling (26). miR-BART15-3p targets the NLR family pyrin domain-containing protein 3 (NLRP3; a member of the inflammasome) and inhibits the synthesis of IL-1β and IL-18 (25, 36). miR-BART2-5p maintains tumor cell survival by downregulating the major histocompatibility complex (MHC) class I polypeptide-related sequence B (MICB) recognized by the natural killer group 2 member D receptor present on NK cells (31, 37).

The BART miRNA coding sequence from the Akata strain was inserted into the B95-8 strain to restore the deleted region (30). As compared to the parental B95-8 strain, the restored B95-8 strain showed a decrease in the activity of nuclear factor kappa light chain enhancer of activated B cells (NF-κB) (30).

### Inhibition of Host Adaptive Immunity by EBV miRNAs

EBV miRNAs also suppress host adaptive immunity (Table 1) (10). BART miRNAs regulate adaptive immunity during latent and lytic infection. In comparison, BHRF1 miRNAs regulate adaptive immunity only during lytic infection. miR-BART1-3p, miR-BART2-5p, miR-BART10-3p, miR-BART22-3p, and miR-BHRF1-2-3p suppress the expression of IL-12B in infected cells. There is a significant decrease in the levels of IL-12 in EBV-infected B lymphocytes that impairs the differentiation of CD4^+^ T cells into T helper 1 (Th1) cells, thereby abrogating host immune response. Thus, there is a reduction in cytotoxic T cells specific for the EBV antigens (22, 32, 38).

miR-BHRF1-3-5p and miR-BART17-5p target transporter associated with antigen processing 2 (TAP2) that transports antigenic peptides to MHC class I molecules, thus, viral antigen presentation is impaired in CD8^+^ T cells (24). EBV miRNAs also target genes involved in antigen processing, such as cystatin-B (CSTB), asparagine endopeptidase (LGMN), and gamma-interferon-inducible lysosomal thiol reductase (IFI30). Thus, antigen presentation is reduced in EBV-infected cells. Similarly, immunodeficient mice transplanted with human hematopoietic stem cells and infected with EBV possess proliferating EBV-infected B lymphocytes owing to reduced immune recognition by the human CD8^+^ T cells (39).

The B cell receptor (BCR) that mediates adaptive immunity as well as lytic infection in EBV-infected B lymphocytes is inhibited by miR-BHRF1-2-5p and miR-BART2-5p (34). miR-BART18-5p targets mitogen-activated protein kinase kinase kinase 2 (MAP3K2) that is a downstream effector in BCR signaling (28). The miR-BHRF1 cluster is considered to suppress constitutive lytic infection and adaptive immunity.

Lymphocyte antigen 75 (LY75) is a membrane protein that is expressed on dendritic cells and induces differentiation of Th0 to Th1 cells. miR-BART1-5p (transferred by exosomes) targets LY75 in dendritic cells suppressing Th1 cell differentiation (23).

The roles of EBV miRNAs in suppressing innate and adaptive immunity has been summarized in Figure 1B.

## Host miRNA-Mediated Evasion of the Immune System by EBV-Infected Cells

EBV exploits host miRNAs to escape from the immune system. EBNA2 is a viral protein that expressed during type III latency and upregulates miR-21, that subsequently downregulates myeloid differentiation factor 88 (MyD88) and IL-1 receptor-associated kinase 1 (IRAK1) (40). The miR-17-92 cluster, which is essential for the differentiation of immune cells, is highly expressed in EBV-positive tumors, such as NPC (41) and DLBCL (42). High expression of miR-17-92 in B cells, T cells, NK cells, macrophages, and dendritic cells is known to inhibit cellular differentiation and function (43).

In EBV-infected B lymphocytes, viral LMP1 activates NF-κB signaling and host miR-155. But miR-155 attenuates NF-κB signaling to stabilize persistent infection (44). The miR-155 also targets suppressor of cytokine signaling 1 (SOCS1), a suppressor of the JAK-STAT signal (45). Though miR-155 is upregulated, strong expression of SOCS1 can be observed in EBV-infected cells (46). Simultaneous upregulation of SOCS1 and miR-155 has become an important controversy for researchers who study herpesviruses (47). It might be possible that miR-155 may target another gene expressed higher than SOCS1 in NPC cells.

## Depletion of Viral miRNAs in EBV-Associated Tumors

In EBV-infected epithelial tumor cells, BART miRNAs are highly expressed and help in evading immune recognition (10). However, the BART miRNA clusters are frequently depleted in virus causing chronic active EBV infection, ENKL, and DLBCL (48, 49). BART miRNA were found lacking in 71% of DLBCL cases (49). On the other hand, DLBCL patients with high BART miRNA expression in the blood showed worse prognosis than patients with low expression (50). Although high expression of BART miRNAs is possibly important for malignant transformation of lymphoma, it may be disadvantageous for lymphoma cells survival by escaping immune surveillance.

Similarly, LMP1 is expressed in all the early NPC tumor cells and contributes to pleiotropy in NPCs (51). However, once NF-κB signaling is sufficiently active in NPC tumor cells, LMP1 is frequently downregulated (52).

As mutations and/or promoter methylation accumulate in the host genome, the presence of the viral genome may no longer be required for the growth of the tumor cell. In such a situation, carrying large EBV genomes may be a burden for host cells; thus, cells harboring the defective, but oncogenic, EBV genome may proliferate faster than cells infected with EBV having the complete genome. Alternatively, the increased levels of BART miRNAs may repress the expression of genes important for survival of EBV-positive cells. Therefore, further investigation is necessary to discern the physiological significance of BART miRNAs in EBV-positive tumor cells.

## Single Nucleotide Polymorphisms (SNPs) in the Viral miRNA Promoters

BART miRNAs are important in evading the immune system and inhibiting apoptosis. However, multiple BART miRNAs frequently target the same gene to induce a high level of repression (16, 17). This hinders the development of efficacious drugs that must target each BART miRNA in EBV-associated malignancies. Thus, blocking the BART miRNA promoters could be a better strategy to target all the necessary miRNAs (53, 54). We have recently reported a characteristic SNP in the promoter of BART that increases BART promoter activity. This SNP is frequently detected in EBV-associated NPC with an odds ratio of 5.7 (55). Therefore, studying the promoter of BART and the SNPs associated with it can help develop strong candidates that suppress BART transcription.

## Conclusion

EBV uses miRNAs to switch between lytic and latent infection. This helps maintain EBV infection and evade recognition of EBV by the host immune system by reducing viral gene (antigenic) expression. EBV miRNAs also target and suppress genes involved with host immunity. This oncogenic virus also exploits miRNAs for malignant transformation. Exosomes secreted from EBV-infected B lymphocytes contain a large amount of host and viral miRNAs that are transferred to epithelial cells (56). Therefore, miRNAs derived from EBV-infected cells may affect infected and uninfected host cells. Finally, future research may help treat EBV-associated malignancies by developing anti-tumor drugs that inhibit BART promoter activity.

## Author Contributions

HI wrote the manuscript. HK, AK, and YK prepared the table and figures. HY complied the manuscript.

### Conflict of Interest

The authors declare that the research was conducted in the absence of any commercial or financial relationships that could be construed as a potential conflict of interest.
