# Corrigendum: Role of Viral and Host microRNAs in Immune Regulation of Epstein-Barr Virus-Associated Diseases

**DOI:** 10.3389/fimmu.2020.00498

**Published:** 2020-04-03

**Authors:** Hisashi Iizasa, Hyoji Kim, Andy Visi Kartika, Yuichi Kanehiro, Hironori Yoshiyama

**Affiliations:** Department of Microbiology, Faculty of Medicine, Shimane University, Shimane, Japan

**Keywords:** microRNAs, herpes virus, immune evasion, BART miRNA, BHRF miRNA

In the original article, there was a mistake in Table 1 as published. The heading “**miR-BARTs**” has been removed and the heading “**BART miRNA cluster 1&2**” has been replaced with “**Others**.” In addition, reference 22 has been added to rows 3 and 10 under “**Others**.” The corrected Table 1 appears below.

**Table 1:** EBV miRNAs targeting host immune related genes.

**miRNA**	**Host targeting genes**	**Target**	**References**	**EBV infected cells**
**BART miRNAs cluster 1**				
miR-BART1-3p	IL12B	Adaptive immunity	(22)	LCL
miR-BART1-5p	LY75	Adaptive immunity	(23)	LCL
miR-BART1-5p, 3p	IFI30	Adaptive immunity	(24)	LCL
miR-BART15-3p	NLRP3	Innate immunity	(25)	BL
miR-BART16-5p	CREBBP	Innate immunity	(26)	BL, EBVaGC
miR-BART17-5p	TAP2	Adaptive immunity	(24)	LCL
miR-BART6-3p	RIG-I	Innate immunity	(27)	NPC
**BART miRNAs cluster 2**				
miR-BART18-5p	MAP3K2	BCR signals, Adaptive Immunity	(28)	BL
miR-BART8-5p, 3p	STAT1	Innate immunity	(29)	NKTL
miR-BART22-3p	IL12B	Adaptive immunity	(22)	LCL
miR-BART10-3p	IL12B	Adaptive immunity	(22)	LCL
miR-BART20-5p	IFNG	Innate immunity	(29)	NKTL
**Others**				
miR BARTs	NF-kB signal	Innate immunity	(30)	NPC
miR-BART2-5p	MICB	Immunoreaction against NK cell	(31)	LCL
miR-BART2-5p	IL12B	Adaptive immunity	(22, 32)	LCL
miR-BART2-5p	CSTB	Adaptive immunity	(24)	LCL
miR-BART2-5p	LGMN	Adaptive immunity	(24)	LCL
miR-BHRF1	IL-1R1	Innate immunity	(33)	LCL
miR-BHRF1-2-5p	MALT1	BCR signals, Adaptive Immunity	(34)	LCL, DLBL
miR-BHRF1-2-5p	GRB2	BCR signals, Adaptive Immunity	(34)	LCL, DLBL
miR-BHRF1-2-5p	PAG1	BCR signals, Adaptive Immunity	(34)	LCL, DLBL
miR-BHRF1-2-3p	IL12B	Adaptive immunity	(22, 32)	LCL
miR-BHRF1-2-3p	CSTB	Adaptive immunity	(24)	LCL
miR-BHRF1-2-3p	TAP2	Adaptive immunity	(24)	LCL
miR-BHRF1-3-5p	CXCL-11	Innate immunity	(35)	BL, DLBCL

*LCL, Lymphoblastoid cell lines; BL, Burkitt lymphoma; NPC, Nasopharengeal carcinoma; EBVaGC, EBV asspciated gastridc carcinoma; NKTL, NK/T lymphoma; DLBCL, Diffuce large B cell lymphoma*.

The authors apologize for this error and state that this does not change the scientific conclusions of the article in any way. The original article has been updated.

